# Engineering Phosphogypsum into a Fluorescent Nanosensor Based on Functionalized Nano-CaCO_3_ for Selective Hg^2+^ Detection

**DOI:** 10.3390/ma19153271

**Published:** 2026-08-03

**Authors:** Han Mo, Qingwei Liu, Nenghao Wang, Wentao Zhou, Mingyang Sun, Wuyi Wang, Xiao Huang, Huali Zhang

**Affiliations:** School of Chemical and Environmental Engineering, Wuhan Institute of Technology, Wuhan 430205, Chinazhanghl413@126.com (H.Z.)

**Keywords:** phosphogypsum, nano-calcium carbonate, surface modification, fluorescent nanosensor

## Abstract

Phosphogypsum (PG) is a major solid by-product generated during wet-process phosphoric acid production. Its stockpiling poses significant environmental risks due to the potential leaching of harmful substances. Therefore, conversion of PG into high-value-added products is urgent. In this work, nano-calcium carbonate (Nano-CaCO_3_) was synthesized from PG via a phase-transfer precipitation method. The surface of the as-prepared product was then modified for Hg^2+^ detection. Initially, the nanoparticle surface was functionalized with (3-aminopropyl)triethoxysilane (APTES), followed by grafting with a synthesized rhodamine B-based derivative (RhoB-NCS). The acquired product was denoted as CaCO_3_@RhoB-NCS. Physical characterization demonstrated its successful fabrication. The product is an effective sensor for Hg^2+^ detection, exhibiting enhanced fluorescence emission at 600 nm with a detection limit as low as 1.0 × 10^−6^ M. Moreover, the CaCO_3_@RhoB-NCS was used as a filler for Hg^2+^ test paper. This work demonstrates a promising approach for valorizing solid waste into high-value-added product.

## 1. Introduction

Phosphogypsum (PG) is a major solid waste generated from the wet-process phosphoric acid industry, with a substantial global annual output. Approximately 5 tons of PG are produced per ton of phosphoric acid manufactured. Worldwide, PG stockpiles have accumulated to an estimated 6 billion tons, while annual generation continues to rise by approximately 150 million tons. The PG inventory is 6 billion tons in 2025, and it is expected that the global inventory of PG will double by 2045 [1]. Despite some existing applications in construction materials and other sectors, the overall utilization rate of PG remains low [1]. Currently, PG is mainly stored in stockpiles which not only occupy land but also pose risks of leaching soluble phosphorus, fluorine, and trace heavy metals via rainwater, leading to secondary pollution of soil and water bodies [2,3]. Therefore, developing high-value-added resource recovery technologies to converting PG into valuable products is crucial to addressing these environmental challenges and advancing the circular economy [4,5].

Various high-value-added products can be realized via the element utilization of PG. Among them, nano-calcium carbonate represents a highly promising pathway [6,7]. As an important functional inorganic filler, nano-calcium carbonate is extensively used in plastics, rubber, coatings, paper, and biomedical fields [8]. Currently, the industrial production of nano-calcium carbonate mainly relies on natural limestone, consuming non-renewable mineral resources. Using PG as an alternative to limestone can not only consume solid waste but also create economic value [9]. Various processes such as carbonation and double decomposition have been explored. Among them, the “phase-transition precipitation method” offers the possibility of producing high-purity nano-calcium carbonate by effectively separating calcium ions from impurity ions (e.g., phosphate and fluoride) in PG [10,11,12,13]. Studies show that this method can produce uniform calcite-type nano-calcium carbonate particles with sizes around 30 nm. Similarly, Yang et al. [14] have successfully synthesized morphology-controlled calcium carbonate microparticles from calcium-containing solid wastes, such as PG and phosphate tailings, via a route combining phase-transition with carbonation.

Nano-calcium carbonate plays a vital role in many fields. However, untreated nano-calcium carbonate possesses a highly hydrophilic surface and tends to agglomerate strongly due to its high surface energy. It also lacks specific chemical functionality, which limits its application in high-end areas. Surface organic modification is a key strategy to overcome this limitation [15,16,17]. Modification with silane coupling agents, such as 3-aminopropyltriethoxysilane (APTES) [18], can introduce organic functional groups such as amino groups onto the particle surface, which effectively improves its dispersibility and provides reactive sites for further functionalization [19,20]. Current research frontiers have moved beyond simple homogeneous modification to constructing “Janus” particles with asymmetric chemical properties. For instance, Khin et al. [21] successfully prepared Janus-type nano-calcium carbonate particles with tunable amphiphilic balance. They achieved this through an ingenious strategy involving wax emulsion protection and a two-step surface modification process, demonstrating a strong potential of surface modification techniques in endowing materials with novel properties. Previous work gives us an inspiration that PG can be constructed into a high-performance functional material by precisely engineering the surface of phosphogypsum-derived nano-calcium carbonate [22].

Among various fluorescent probe molecules, rhodamine B and its derivatives are ideal for constructing “turn-on” fluorescent sensors due to their excellent optical properties and high quantum yield [23,24]. Their spirolactam structure can open upon binding with specific ions like Hg^2+^, producing strong fluorescence and color changes with high sensitivity [25,26]. Recently, researchers have focused on immobilizing rhodamine-based probes onto various nanocarriers such as mesoporous silica and carbon dot composites to enhance sensor stability and reusability. For instance, Miao et al. [27] constructed a ratiometric fluorescent sensor by bonding rhodamine hydrazide to carbon dot surfaces via amidation. Anna et al. [28] systematically studied the immobilization of rhodamine derivatives onto mesoporous silica carriers using APTES as a linker for Hg^2+^ detection, investigating the effects of immobilization methods and loading amounts on performance. These works demonstrate that combining functional molecules with solid carriers is an effective strategy for building practical sensing platforms [29].

In this work, PG was utilized as a raw material to fabricate CaCO_3_@RhoB-NCS particles with Hg^2+^ recognition functionality. Initially, well-dispersed nano-calcium carbonate was prepared from industrial PG via an optimized phase-transition–precipitation method. The surface of the nano-CaCO_3_ was then functionalized with APTES through silanization and a rhodamine derivative, serving as a specific probe for Hg^2+^ recognition, was synthesized and covalently grafted onto the amine-functionalized nano-CaCO_3_ surface. Physical characterization demonstrated the successful fabrication of CaCO_3_@RhoB-NCS. The product exhibits an enhanced fluorescence emission at 600 nm with a detection limit as low as 1.0 × 10^−6^ M. This strategy not only opens a new avenue for the high-value utilization of PG but also offers a promising platform for developing low-cost, high-performance sensors for heavy metal ions detection.

## 2. Experimental

### 2.1. Sample Preparation

Nano-CaCO_3_ was synthesized using the Phase-Transition Precipitation Method [13]. Briefly, 50 g of KCl was added to 300 mL of deionized water under stirring. Subsequently, 5 g of PG was introduced and its chemical composition is presented in Table 1, and the mixture was stirred at room temperature for 40 min. The solid and liquid phases were then separated to obtain a phase-transition solution containing calcium ions. To this solution, 70 mL of KOH aqueous solution (1 mol/L) and citric acid (5 wt%) were added. The reaction was carried out at 40 °C while CO_2_ was introduced for 30 min. The final product, Nano-CaCO_3_, was obtained by filtration and dried for 24 h.

Rhodamine hydrazide (RhoB-NH_2_) was synthesized following the reported procedure [30,31]. Briefly, 1.96 g of rhodamine B (RhoB) and 4 mL of 80% hydrazine hydrate were dissolved in ethanol. The solution was refluxed at 90 °C for 24 h under stirring. After cooling, the mixture was transferred to a flask, and the organic solvent was evaporated under reduced pressure. The residue was extracted with 100 mL of dichloromethane and 200 mL of water, and this extraction process was repeated twice. The combined organic layers were dried over anhydrous Na_2_SO_4_, then concentrated by evaporation, and the crude product was dried under vacuum at 50 °C for 12 h to obtain rhodamine hydrazide.

Rhodamine B isothiocyanate (RhoB-NCS) was synthesized according to the reported literature procedure [30,32]. Rhodamine hydrazide (0.26 g) and 1,4-phenylene diisothiocyanate (0.11 g) were dissolved in 50 mL of dimethylformamide (DMF). The reaction mixture was heated at 80 °C under stirring for 12 h. The crude product was purified by silica gel column chromatography to afford the target compound RhoB-NCS as a red solid. ^1^H NMR (400 MHz, CDCl_3_) δ 7.99 (s, 1H), 7.62 (d, J = 29.2 Hz, 3H), 7.06 (d, J = 35.2 Hz, 5H), 6.40 (d, J = 62.8 Hz, 6H), 3.34 (s, 8H), 1.16 (s, 12H).

The PG-derived Nano-CaCO_3_ was modified to obtain the final composite material of CaCO_3_@RhoB-NCS. First, 2 g of Nano-CaCO_3_ was dispersed in 100 mL of ethanol via ultrasonication for 10 min. Then, 2 g of 3-aminopropyltriethoxysilane (APTES) was added to the suspension. The mixture was stirred and heated at 60 °C for 2 h. The resulting solid was collected by filtration, washed 2–3 times with anhydrous ethanol, and dried for 24 h to yield the intermediate product, CaCO_3_@APTES.

A total of 0.75 g of CaCO_3_@APTES was ultrasonically dispersed in 50 mL of dimethylformamide (DMF) for 10 min. Subsequently, 0.15 g of the synthesized rhodamine derivative (RhoB-NCS) was added to the dispersion. The reaction was carried out at 80 °C under stirring for 24 h. The final product, CaCO_3_@RhoB-NCS, was obtained by filtration and dried.

### 2.2. Characterization

The chemical composition of the sample was analyzed using an X-ray fluorescence spectrometer (XRF, Bruker S8 TIGER, Bruker, Karlsruhe, Germany) operated at 50 kV and 60 mA, with a measurement time of 30 s per element. The morphology of the Nano-CaCO_3_ derived from PG was characterized by scanning electron microscopy (SEM, SU8600, Hitachi High-Technologies, Tokyo, Japan) at an accelerating voltage of 3.00 kV and a working distance of 8.6 mm. The powder was dispersed in ethanol, dropped onto a silicon wafer, dried, and then sputter-coated with Pt for 60 s prior to imaging. The phase of the CaCO_3_@RhoB-NCS particles was investigated using an X-ray diffractometer (XRD, X’Pert^3^ MRD, PANalytical, Almelo, The Netherlands) with Cu Kα radiation (λ = 1.5406 Å) over a 2θ range of 5–90°, at a step size of 0.04° and a scan speed of 2°/min. Changes in the surface functional groups of the Nano-CaCO_3_ particles were determined by Fourier transform infrared spectroscopy (FTIR, Nicolet iS5, Thermo Fisher Scientific, Madison, WI, USA) in the range of 400–4000 cm^−1^, with a resolution of 3.818 cm^−1^ and 32 scans. Samples were prepared using the KBr pellet method. The element states were analyzed by X-ray photoelectron spectroscopy (XPS, ESCALAB 250Xi, Thermo Fisher Scientific, East Grinstead, UK). ^1^H NMR spectra were recorded on a spectrometer using tetramethylsilane (TMS, AV400, Bruker, Rheinstetten, Germany) as an internal standard.

## 3. Results

### 3.1. Characterization of Unmodified and Surface-Modified Nano-CaCO_3_

**Figure 1:**
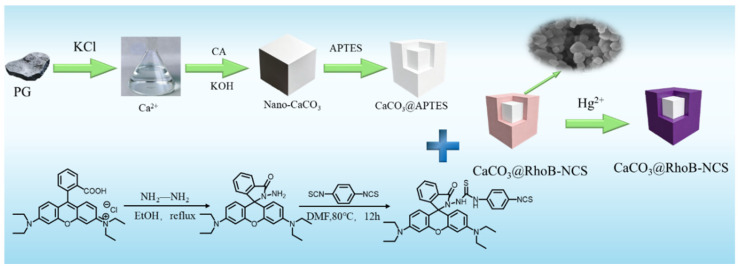
Schematic of the synthesis process for CaCO_3_@RhoB-NCS.

**Figure 2:**
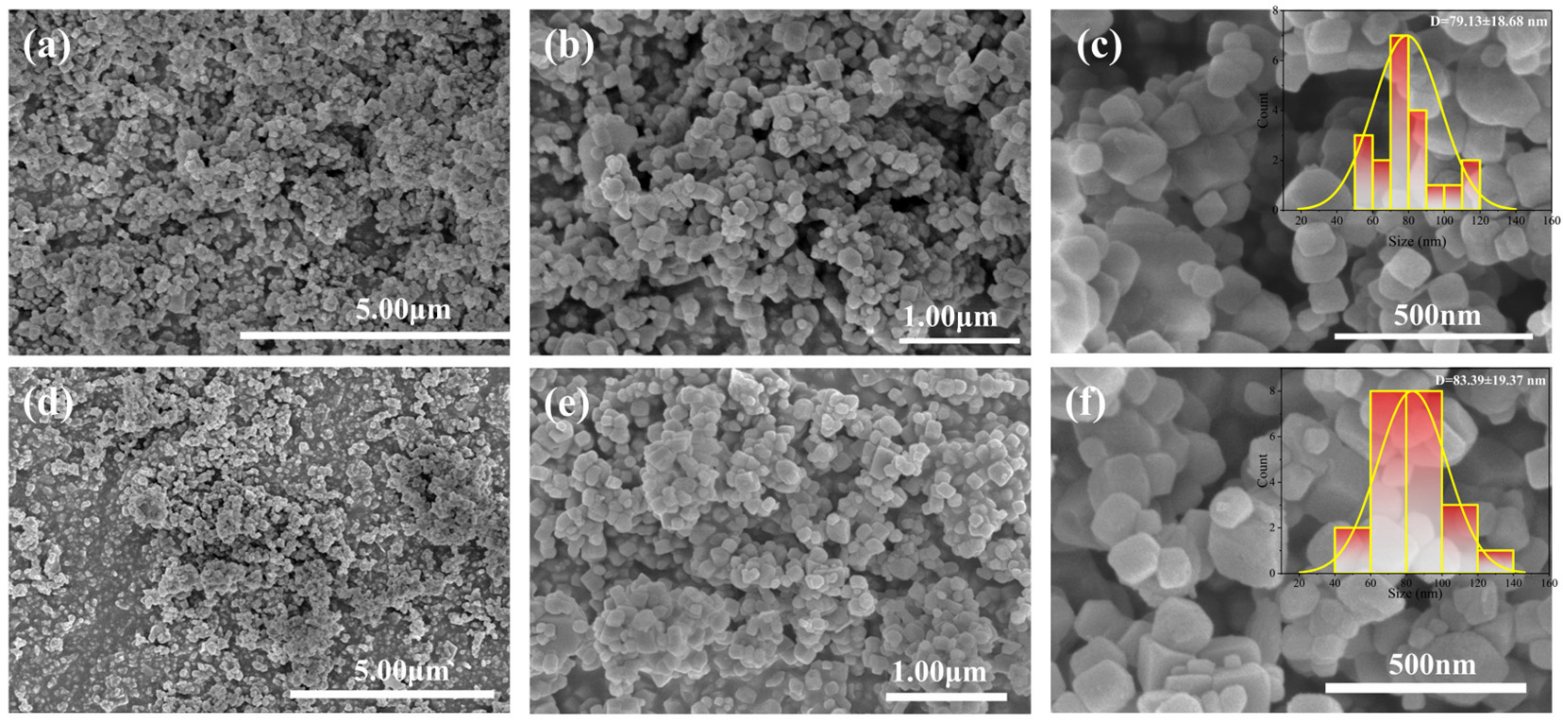
SEM images of Nano-CaCO_3_ and CaCO_3_@RhoB-NCS. (**a**) Nano-CaCO_3_, scale bar: 5 µm; (**b**) Nano-CaCO_3_, scale bar: 1 µm; (**c**) Nano-CaCO_3_, scale bar: 500 nm; (**d**) CaCO_3_@RhoB-NCS, scale bar: 5 µm; (**e**) CaCO_3_@RhoB-NCS, scale bar: 1 µm; (**f**) CaCO_3_@RhoB-NCS, scale bar: 500 nm.

CaCO_3_@RhoB-NCS was synthesized as illustrated in Figure 1. Initially, SEM images were given to study the morphology of the as-prepared sample. Figure 2a–c show that the obtained Nano-CaCO_3_ sample consists of relatively dispersed cubic particles with particle size of about 79 nm. Furthermore, we studied the effect of organic modification on the morphology. Figure 2d–f present the morphology of the Nano-CaCO_3_ after surface organic modification. The morphology of Nano-CaCO_3_ after modification with APTES and RhoB-NCS shows an ignored change with particle size distribution at 83 nm.

**Figure 3:**
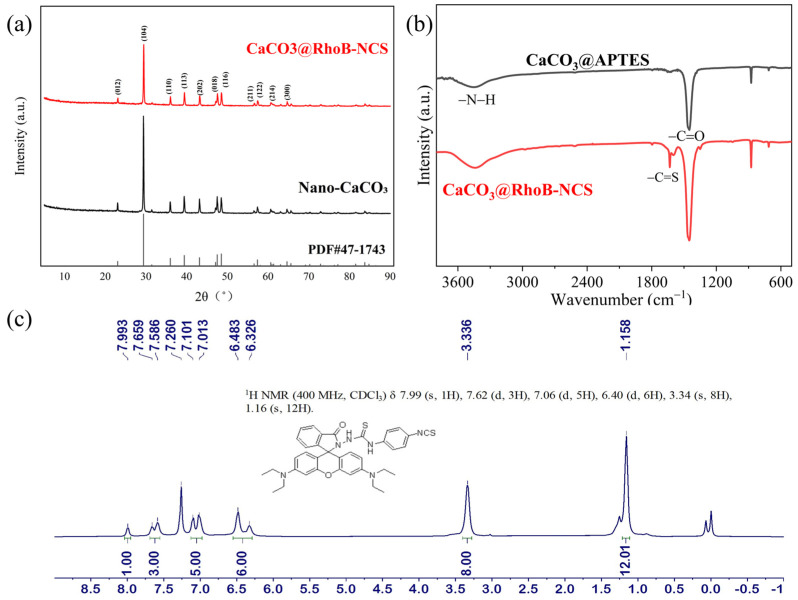
(**a**) XRD patterns of Nano-CaCO_3_ and CaCO_3_@RhoB-NCS; (**b**) FT-IR spectra of CaCO_3_@APTES and CaCO_3_@RhoB-NCS; and (**c**) 1H NMR spectrum of RhoB-NCS.

XRD was employed to investigate the crystal structure of the synthesized products. As shown in Figure 3a, all distinct diffraction peaks observed for both the pristine Nano-CaCO_3_ and the functionalized CaCO_3_@RhoB-NCS composite can be readily indexed to the calcite phase of calcium carbonate, which is in agreement with the standard data (JCPDS Card No. PDF#47-1743). The characteristic peaks at 2θ values of 23.1°, 29.4°, 36.0°, 39.4°, 43.2°, 47.5°, and 48.5° correspond to the (012), (104), (110), (113), (202), (018), and (116) crystal planes of calcite, respectively [33]. The presence of the highly crystalline (104) peak as the main reflection confirms the successful transformation of PG into well-structured calcite. Compared to the unmodified Nano-CaCO_3_, the XRD pattern of CaCO_3_@RhoB-NCS shows no peak shift or new phases, indicating that the surface grafting process with APTES and the rhodamine derivative does not alter the intrinsic crystal lattice of the calcite core.

FT-IR spectra of the samples obtained during the sequential modification with APTES and RhoB-NCS are shown in Figure 3b. For the sample modified with APTES, peaks at 876.06 cm^−1^, 3452.93 cm^−1^, and 1454.77 cm^−1^ are observed, which are attributed to the ν_2_ bending vibration of carbonate in calcite, and the stretching vibrations of amino (NH_2_) and carbonate (CO_3_^2−^) groups, respectively [30]. This confirms the successful grafting of APTES onto the Nano-CaCO_3_ surface. After further grafting of RhoB-NCS onto the APTES-modified sample, peaks at 876.11 cm^−1^, 3437.19 cm^−1^, and 1451.86 cm^−1^ remain. Additionally, new peaks appear at 1631.94 cm^−1^ and 1599.40 cm^−1^, which can be assigned to vibrations of C=S and C–N groups. The presence of the C=S vibration indicates the successful reaction and grafting of RhoB-NCS [30].

The successful synthesis of the target molecule RhoB-NCS was confirmed by ^1^H NMR spectroscopy (400 MHz, CDCl_3_) of the purified red solid product, as shown in Figure 3c. The characteristic signals are assigned as follows: the singlet at δ 7.99 (s, 1H) corresponds to the aromatic proton adjacent to the lactam nitrogen of the rhodamine core. Multiplets in the range of δ 7.65–7.01 ppm (integral corresponding to approximately 15H) are attributed to the aromatic protons of the rhodamine xanthene ring and the newly introduced phenyl ring. Notably, the signals at δ 6.48 and 6.32 ppm are characteristic of the xanthene ring protons, indicative of the opened spirolactam form of rhodamine. The signal at δ 3.34 (s, 8H) corresponds to the eight equivalent methylene protons of the –N–CH_2_–CH_2_– groups linked to the tertiary amine nitrogen. The sharp singlet at δ 1.16 (s, 12H) is assigned to the twelve equivalent methyl protons of the four terminal –CH_3_ groups. The chemical shifts, peak multiplicities, and integrated areas of all observed proton signals are fully consistent with the expected molecular structure of RhoB-NCS [30]. Furthermore, no significant characteristic impurity peaks from the starting materials (rhodamine hydrazide or 1,4-phenylene diisothiocyanate) were detected, indicating high purity of the product suitable for subsequent surface modification reactions.

**Figure 4:**
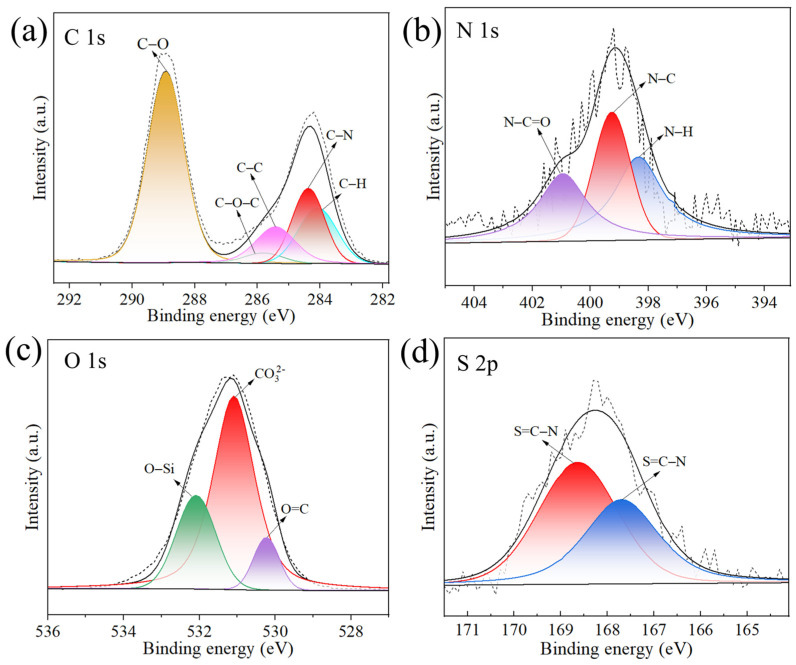
(**a**) C 1s, (**b**) N 1s, (**c**) O 1s, and (**d**) S 2p core-level spectra of CaCO_3_@RhoB-NCS.

**Figure 5:**
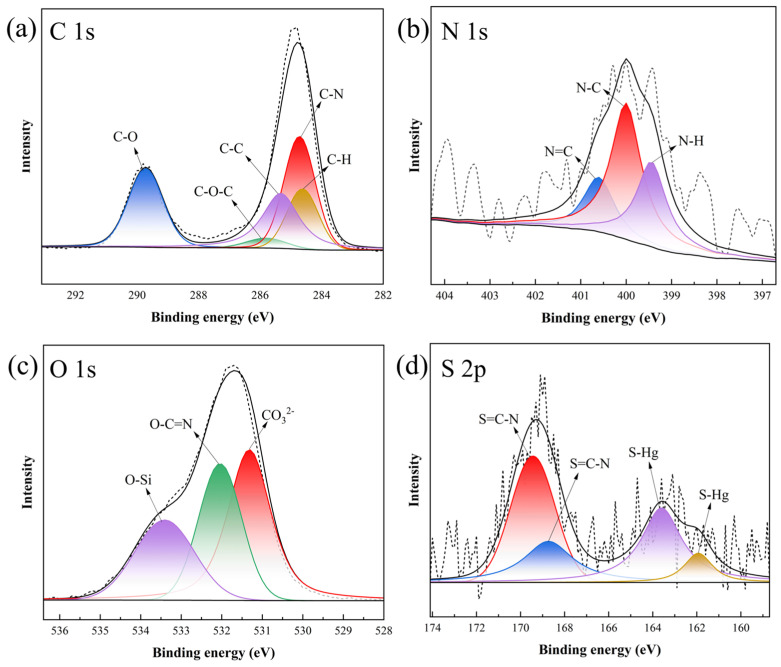
(**a**) C 1s, (**b**) N 1s, (**c**) O 1s, and (**d**) S 2p core-level spectra of CaCO^3^@RhoB-NCS + Hg^2+^.

Figure 4 and Figure 5 show the XPS spectra of CaCO_3_@RhoB-NCS before and after its reaction with Hg^2+^, respectively. All binding energies were calibrated by referencing the C 1s peak of adventitious carbon to 284.8 eV. A Shirley-type background was subtracted from each core-level spectrum prior to peak fitting. The peak shapes were modeled using a Gaussian–Lorentzian sum function with 30% Lorentzian contribution [GL (30)]. During the fitting procedure, the full width at half maximum (FWHM) values of peaks belonging to the same chemical element were constrained to be comparable. For the S 2p doublet, the spin-orbit splitting was fixed at 1.18 eV with an area ratio of 2:1 (2p_3_/_2_:2p_1_/_2_). The fitting was iterated until a satisfactory convergence (χ^2^ minimization) was achieved for all spectra. In the C 1s spectrum of the composite (Figure 4a), the high intensity of the C-O bond is attributed to the underlying CaCO_3_. The N 1s spectrum of CaCO_3_@RhoB-NCS (Figure 4b) exhibits two distinct components, assignable to the –NH_2_ group from APTES and the N–C=O moiety from the grafted RhoB-NCS, respectively, thereby confirming the successful covalent immobilization of the rhodamine probe. Correspondingly, the dominant peak in the O 1s spectrum (Figure 4c) originates from the CO_3_^2−^ in CaCO_3_. The simultaneous presence of signals for C-O-C, C-N, O-Si, and C=O bonds in the C 1s and O 1s spectra confirms the successful surface modification. This indicates that RhoB-NCS primarily resides on the Nano-CaCO_3_ particle surface. Furthermore, the appearance of a new S 2p signal corresponding to the S=C-N bond (Figure 4d) provides direct evidence for the successful grafting of RhoB-NCS.

XPS analysis was then performed on the composite after the addition of Hg^2+^ (Figure 5). The C 1s spectrum of CaCO_3_@RhoB-NCS after Hg^2+^ exposure (Figure 5a) remains dominated by the carbonate peak along with C–C and C–N contributions, indicating that the calcite core and the organic backbone are preserved during Hg^2+^ sensing. The absence of any significant peak shifts or new carbon species confirms that the Hg^2+^-triggered response primarily involves the heteroatoms (N, O, S) of the rhodamine moiety rather than altering the carbon bonding environment. Comparison of the N 1s (Figure 5b) and O 1s (Figure 5c) XPS spectra before and after the reaction with Hg^2+^ reveals that the original N-C=O and O=C bonds are replaced by N=C and O–C=N bonds. This change confirms a reaction between Hg^2+^ and the grafted RhoB-NCS, which is responsible for the observed color change. The mechanism involves the cyclization of the thiosemicarbazide moiety on the rhodamine derivative upon reacting with Hg^2+^, forming a cyclic 1,3,4-oxadiazole structure [32]. Additionally, the appearance of an S-Hg signal in Figure 5d further supports this mechanism. It suggests that sulfur is released during the reaction and subsequently binds to the Hg^2+^ on the particle surface.

### 3.2. Fluorescence Properties

**Figure 6:**
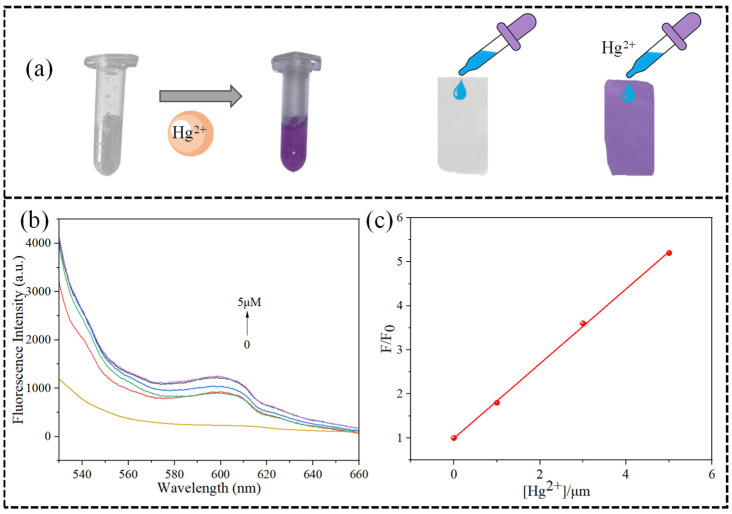
(**a**) Color response, (**b**) fluorescence emission spectra, and (**c**) fluorescence intensity at λex = 500 nm of CaCO_3_@RhoB-NCS nanoparticle suspension upon addition of Hg^2+^ at different concentrations.

Figure 6 illustrates the color and fluorescence changes of the CaCO_3_@RhoB-NCS particles in aqueous media upon exposure to Hg^2+^. As shown in Figure 6a, the addition of Hg^2+^ to a suspension containing the particles induced a distinct color change from light pink to purple, visible to the naked eye. To demonstrate a practical application, two simple test strips were prepared using CaCO_3_@RhoB-NCS particles as the active filler. A droplet of Hg^2+^ solution was applied to one strip, and a droplet of deionized water was applied to the other as a control. After complete evaporation, a clear color difference was observed, as shown in the figure. These results confirm a potential application of the phosphogypsum-derived CaCO_3_@RhoB-NCS nanoparticles for mercury ion detection.

The fluorescence response was investigated to evaluate the sensing performance toward Hg^2+^. From the fluorescence spectra in Figure 6b, the CaCO_3_@RhoB-NCS nanoparticles exhibit a strong emission band centered at 600 nm (λex = 500 nm). The fluorescence intensity gradually increased with the rising concentration of Hg^2+^. Figure 6c plots the relationship between fluorescence intensity and Hg^2+^ concentration. A good linear correlation was established within the range of 0 to 5 μM. These findings indicate that the CaCO_3_@RhoB-NCS nanoparticles possess high sensitivity towards Hg^2+^. The calculated limit of detection (LOD) reaches 1.0 × 10^−6^ M.

**Figure 7:**
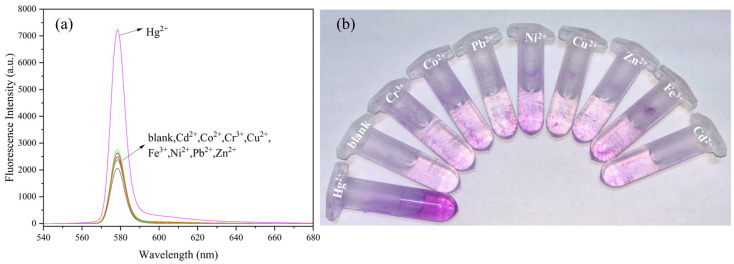
(**a**) Fluorescence emission spectra; (**b**) color response.

To evaluate the selectivity of CaCO_3_@RhoB-NCS NPs for various metal ions including Fe^3+^, Pb^2+^, Cr^3+^, Ni^2+^, Co^2+^, Zn^2+^, Cd^2+^ and Cu^2+^, the fluorescence changes induced by equal concentrations of these metal ions were measured in a water/nitric acid solution (Figure 7). In Figure 7a, a significant enhancement in fluorescence intensity was observed only in the presence of Hg^2+^, while other metal ions did not cause any significant changes under identical conditions. In addition, no significant color changes were observed by the naked eye for other metal ions except mercury ions in water/nitric acid media (Figure 7b). These results indicate that CaCO_3_@RhoB-NCS NPs possess a high selectivity for Hg^2+^.

To further evaluate the competitiveness of the as-prepared CaCO_3_@RhoB-NCS nanosensor, its sensing performance was compared with recently reported rhodamine-based Hg^2+^ sensors. It should be noted that, to the best of our knowledge, no rhodamine-based Hg^2+^ sensor fabricated from industrial solid waste has been reported to date, which makes a direct comparison with sensors sharing an identical raw-material origin infeasible. Nevertheless, a comparison with rhodamine-based sensors employing different carriers or operating in homogeneous phases remains instructive.

The detection limit of CaCO_3_@RhoB-NCS (1.0 × 10^−6^ M) and its linear detection range (0–5 μM) are identical to those of the Fe_3_O_4_@SiO_2_-RhoB-NCS magnetic nanosensor reported by Zeng et al. [30], which employs the same rhodamine derivative (RhoB-NCS) as the recognition probe. This comparable performance confirms that substituting synthetic Fe_3_O_4_@SiO_2_ nanoparticles with waste-derived nano-CaCO_3_ does not compromise the sensing capability. It is also worth noting that some homogeneous rhodamine-based probes, such as the one reported by Zhu et al. [24], are able to achieve a lower detection limit (3.0 × 10^−8^ M). However, homogeneous probes inherently suffer from difficulties in separation, recovery, and reuse, which limit their practical applicability. In contrast, solid-supported sensors like CaCO_3_@RhoB-NCS offer distinct advantages in terms of operational convenience, potential recyclability, and the feasibility of fabricating portable test strips—as demonstrated in this work.

From a broader perspective, the most compelling competitiveness of CaCO_3_@RhoB-NCS lies not merely in its detection limit, but in its sustainable “waste-to-resource” strategy. By converting phosphogypsum—a massively stockpiled industrial solid waste—into a functional Hg^2+^ sensing material, this approach simultaneously addresses two environmental challenges: solid waste valorization and heavy metal pollution monitoring. This dual environmental benefit, rooted in the principle of circular economy, fundamentally distinguishes the present work from all previously reported rhodamine-based Hg^2+^ sensors, which rely exclusively on commercially sourced chemical reagents or synthetic nanomaterials.

## 4. Conclusions

In summary, a novel fluorescent nanosensor based on PG-derived nano-CaCO_3_ functionalized with a rhodamine derivative (CaCO_3_@RhoB-NCS) was successfully designed for the selective detection of Hg^2+^ in aqueous media. The as-prepared nanocomposite exhibits a distinct visual color change from light pink to purple upon exposure to Hg^2+^, which is observable by the naked eye, and is accompanied by a significant fluorescence enhancement at 600 nm under irradiation.

Physical characterization confirmed the successful synthesis of well-dispersed nano-CaCO_3_ and the grafting of the RhoB-NCS probe onto the aminosilanized surface. The results indicate that the CaCO_3_@RhoB-NCS nanosensor possesses high selectivity and sensitivity toward Hg^2+^, with a detection limit as low as 1.0 × 10^−6^ M. XPS analysis reveals that the sensing mechanism involves a Hg^2+^-triggered cyclization reaction of the thiosemicarbazide moiety, a process that leads to the formation of a new oxadiazole ring structure and the opening of the spirolactam ring. Furthermore, the practical applicability was demonstrated by fabricating simple test strips, enabling rapid and convenient visual detection of Hg^2+^. The present work indicates that valorizing industrial solid waste into inorganic/organic hybrid nanoparticles serves as a versatile and sustainable strategy for the development of functional sensing platforms. We anticipate that this methodology will find broad utility in environmental monitoring scenarios where cost-effective and portable sensors for heavy metal ions are urgently required.

## Figures and Tables

**Table 1:** Elemental composition analysis of PG.

Ingredients	CaO	SO_3_	SiO_2_	Fe_2_O_3_	P_2_O_5_	K_2_O	Al_2_O_3_	TiO_2_	Loss
Content (wt %)	37.77%	48.68%	10.1%	0.555%	1.17%	0.465%	0.92%	0.13%	0.21%

## Data Availability

The original contributions presented in this study are included in the article. Further inquiries can be directed to the corresponding author.
